# Dose escalation study of an anti-thrombocytopenic agent in patients with chemotherapy induced thrombocytopenia

**DOI:** 10.1186/1471-2407-10-565

**Published:** 2010-10-19

**Authors:** Robert D Levin, MaryAnn Daehler, James F Grutsch, John L Hall, Digant Gupta, Christopher G Lis

**Affiliations:** 1 Cancer Treatment Centers of America® at Midwestern Regional Medical Center, Zion IL, USA; 2 University of Illinois at Chicago, School of Public Health, Chicago IL, USA; 3 Natural Source International, New York, NY, USA

## Abstract

**Background:**

Preclinical studies demonstrated that small chain RNA fragments accelerate the recovery of platelets numbers in animals exposed to high doses of chemotherapeutic drugs. There is anecdotal data supporting the same application in humans. The Phase I clinical trial described here was designed to investigate the relationship between the administration of small chain RNA fragments and the recovery in platelets following Chemotherapy-Induced Thrombocytopenia (CIT).

**Methods:**

Cancer patients with solid tumors that experienced post chemotherapy thrombocytopenia with a nadir of < = 80,000 platelets/ml were eligible for this clinical trial. There were no exclusions based on ECOG status, tumor type, tumor burden or chemotherapeutic agents. Patients received a unique preparation of RNA derived from either E. coli or yeast. Ten patients per group received 20, 40, or 60 mg as a starting dose. Subjects self-administered RNA fragments sublingually on an every other day schedule while undergoing chemotherapy. The dose was escalated in 20 mg increments to a maximum dose of 80 mg if the nadir was < 80,000 platelets/ml at the start of the next cycle. Subjects were treated for three cycles of chemotherapy with the maximum effective dose of RNA fragments. Subjects continued on planned chemotherapy as indicated by tumor burden without RNA fragment support after the third cycle. Subjects kept a diary indicating RNA fragment and magnesium administration, and any experienced side effects.

**Results:**

Patients receiving E. coli RNA fragments demonstrated a more rapid recovery in platelet count and higher nadir platelet count. None of the patients receiving the E. coli RNA fragments required a chemotherapy dose reduction due to thrombocytopenia. The optimal dose for minimizing CIT was 80 mg. Conversely, subjects receiving yeast RNA fragments with dose escalation to 80 mg required a chemotherapy dose reduction per American Society of Clinical Oncology guidelines for grade 3 and 4 thrombocytopenia.

**Conclusions:**

Patients receiving myelosuppressive chemotherapy experienced an improvement in the platelet nadir and shorter recovery time when receiving concurrent E coli RNA fragments, when compared to patients who received yeast RNA fragments. These data indicate that 60 and 80 mg doses of E. coli RNA accelerated platelet recovery. Further clinical investigations are planned to quantify the clinical benefits of the E. coli RNA at the 80 mg dose in patients with chemotherapy induced thrombocytopenia.

**Trial Registration:**

Clinical Trials.gov Identifier: NCT01163110

## Background

Myelosuppressive chemotherapy has the potential to produce life threatening neutropenia, anemia, and thrombocytopenia. All of these conditions compromise therapeutic dosing which impacts survival as well as quality of life. The introduction of recombinant growth factors has enabled oncologists to minimize or prevent the development of treatment-induced anemia and neutropenia, but the management of chemotherapy-induced thrombocytopenia (CIT) remains a major challenge. American Society of Clinical Oncology (ASCO) guidelines recommend dose reduction in chemotherapy following onset of thrombocytopenia despite data showing full dose, on time, chemotherapy leads to reduced tumor burden and better overall survival [1-3]. Patients with CIT experience potentially life threatening complications, delay in treatment, poorer outcomes, and consume inordinate amounts of health care resources for supportive care [4]. Development of an agent that ameliorates CIT would represent a major breakthrough in cancer treatment.

Platelets are anuclear cell particles that are released into the bloodstream by megakaryocytes located in the bone marrow. The differentiation of megakaryocytes is regulated by an intricate interaction of specific cytokines and growth factors [5]. Bone marrow stromal elements are also critical to the differentiation and release of platelets. Cancer chemotherapy often depletes the stem and progenitor cells involved in platelet proliferation, which leads to a diminution of platelets and temporary interruption of platelet production lasting until stromal elements and megakaryocytes regenerate.

There are several experimental agents targeted to prevent thrombocytopenia. These investigational agents are cytokines involved in the differentiation and production of platelets [6]. Currently, the only agent commercially available for prevention of thrombocytopenia is Neumega, a derivative of IL-11. Data on Neumega (Oprelekin) indicates a shorter duration of chemotherapy-induced thrombocytopenia, but the toxicity profile has prevented its widespread introduction into the clinical setting. Overall, the absence of agents that minimize or reverse CIT continues to severely limit many patients' ability to complete the full schedule of chemotherapy at the doses originally prescribed by treating oncologists [1].

This study is a dose escalation trial investigating the anti-thrombocytopenic benefits and safety of single stranded RNA fragments. The short chain RNA fragments are obtained by controlled degradation of prokaryotic RNA with ribonuclease. Beljanski showed that these RNA fragments act as primers for DNA synthesis in vitro and found that variations in the method of degradation yielded different preparations that prime DNA synthesis with distinct tissue specificity. The RNA fragments used in this study, when administered orally, localize in the bone marrow where they appear to prime DNA replication in stem cells resulting in proliferation of white blood cells and platelets. Beljanski et al. reported that these specific RNA fragments were effective in restoring normal levels of circulating platelets following drug induced thrombocytopenia [7,8]. Demonstrating the utility of these specific RNA fragments for prevention and treatment of thrombocytopenia among cancer patients undergoing chemotherapy is especially attractive given the absence of the side effects associated with growth factors and hormones.

This trial investigated the efficacy of two RNA preparations--extracted, purified and fragmented according Beljanski's procedure--to ameliorate CIT: one derived from E. coli and the second from yeast (a eukaryote). RNA molecules are present in any diet and can be considered conditional essential nutrients under conditions of physiological stress [9]. Purified RNA prepared by various methods can be found in nutritional products for hospitalized patients and infant formula [9-11]. This clinical trial evaluated the biologic effects of various doses of these specially prepared 'primer' RNA fragments on platelet numbers in cancer patients who have already developed thrombocytopenia while undergoing chemotherapy.

## Methods

### Patient Selection

This is an onsite study conducted at Cancer Treatment Centers of America^® ^at Midwestern Regional Medical Center, Zion Illinois, USA. Cancer patients with a solid tumor, between the ages of 18 and 80 with CIT with nadir equal to or less than 80,000/ml were eligible to participate in this study. There was no ineligibility based on tumor type, tumor burden, prior treatment history, or chemotherapeutic agents. There were no ECOG performance status restrictions in this investigation, but patients had to have the physiological capacity to undergo myelosuppressive chemotherapy and reliability of self-administration of RNA fragments. After determination of patient eligibility, subjects signed an Informed Consent indicating awareness of the investigational nature of the study and agreement to participate in the first systematic investigation of this agent. Exclusion criteria included a life expectancy of less than three months, a known hypersensitivity to RNA or its metabolic products, or a requirement for administration of therapeutic heparin (manufacturer's recommendations).

### Experimental Agent

The two test agents are mixtures of single-stranded chains of 10 to 80 ribonucleotides in length. Both mixtures have an overall ratio of purine bases to pyrimidine bases (G+A)/(C+U) ranging from 1 to 2.5. One test agent is manufactured by extracting RNA from E. coli strain K 12, while the other test agent is extracted from a yeast strain. Both strains are FDA approved for human consumption. The final product is a lyophilized fraction with mannitol added to give the powder a sweet taste. Both RNA extracts is contained in a unit dose vial as a powder. By manufacturer's recommendation, patients self-administered the agents sublingually in combination with a magnesium supplement every other day to suppress the activity of RNA ribonuclease.

### Study Design

The Institutional Review Board at Cancer Treatment Centers of America^® ^at Midwestern Regional Medical Center (MRMC) reviewed and approved the study protocol in March 2004. After determining eligibility, all patients gave written informed consent before participating in the trial. Due to the myelosuppressive potential of the chemotherapeutic agents, patients were prophylactically treated with colony stimulating agents to maintain white and red blood cell production as per current standards of care [3]. This study was done sequentially; patient recruitment was completed for the E. coli arm before the first patient was recruited for the yeast arm. Each dose was tested in blocks of ten patients starting with 20 mg. As each block was completed the dose was escalated to 40 mg, than to 60 mg. All doses were taken with a magnesium supplement. The magnesium supplement slows the rate of degradation of the RNA fragments in the plasma.

Patient instructions included taking nothing by mouth for 15 minutes pre and post sublingual administration. To prevent digestion of the RNA fragments, sublingual route was chosen. Patients maintained a diary indicating date and time of RNA fragment administration and compliance of magnesium administration. In order to track toxicity, patients documented side effects. Weekly blood counts were evaluated or as needed to evaluate and manage the patients risk of bleeding and patients were transfused per ASCO guidelines [9]. These guidelines indicate that platelet transfusions are required when the patient is bleeding or their platelet levels have fallen below 20000 cells/ml. We used the Common Toxicity Criteria for Thrombocytopenia--Grade 0 is normal; Grade 1 < 150000/ml to 75000; Grade 2 i > 50000 < 75000: Grade 3 > 10000 to < 50000: and Grade 4 is < 10000.

Patients red blood counts were measured before the administration of RNA fragments. To maintain the biological activity of the RNA fragments, patients had blood transfusions whenever their red blood cell level fell below 3 × 10^6 ^cells/ml.

The initial dose of RNA fragments was 20 mg every other day at the patient's convenience. Failure to respond was defined as platelet nadir of 80,000/ml or less or a nadir platelet count lower than prior cycle. Patients that did not achieve platelet levels of greater then 80,000/ml received a dose escalation in 20 mg increments until a dose of 80 mg was reached. Consequently, there were more than ten patients in the 40, 60, and 80 mg doses. Subjects were treated for three cycles of chemotherapy with the maximum effective dose of RNA fragments. Subjects continued on planned chemotherapy as indicated by tumor burden without RNA fragment support after the third cycle.

### Outcome Measures

The objective of this trial was two fold. The first goal is to evaluate the effect of increasing dose of RNA fragments on platelet nadir following chemotherapy and on platelet recovery time. Secondary outcomes were measured by on time chemotherapy administration and the occurrence of platelet/blood transfusions.

### Statistical Methods

Data was collected from patients' diary and medical records for analysis. Descriptive statistical analysis of medians and ranges were performed using the JMP Software Package (SAS, Cary, North Carolina). The Kruskal-Wallis one-way analysis of variance (ANOVA) by ranks, a non-parametric equivalent of one-way ANOVA, was used to determine if there was any statistically significant difference in nadirs or recovery levels by the dose level of the RNA fragments.

## Results

### Patient Characteristics

#### E. coli RNA Arm

Thirty-two patients signed an informed consent form to participate in the E. coli RNA fragment trial. Table 1 describes the baseline demographic and clinical characteristics of the patients in greater detail. The median time between diagnosis of cancer and participation in this trial was 71 weeks (20 to 347 weeks). Four patients had no prior history of anti-cancer chemotherapy before enrollment, but twenty four patients had failed between one to nine prior regimens (mean 2.5 for all patients). Furthermore, these patients were exposed to CIT inducing agents, such as, Carboplatin (n = 9), Adriamycin (n = 10), Cyclophosphamide (N = 9), Cisplatin (n = 6), and Docetoxel (N = 8). Eight patients were treated with mitomycin, which can induce persistent bone marrow damage [12]. Eleven patients had no radiological or clinical signs of metastatic disease, but eleven had metastatic disease to the bone marrow and six of these had disease in flat bones, which could affect platelet production [12], see table 1. Overall, this patient population was heavily pretreated with CIT inducing agents and a significant fraction of these patients experienced Grade 3 CIT (45.7% or 27/59 treatment cycles) at the chemotherapy induced nadir prior to baseline or at dose escalation of the RNA fragments (Table 2).

**Table 1:** Baseline Characteristics of Patients

Characteristic	E. coli (N = 32)	Yeast (N = 31)
Age		
Median	52	55.7
Range	35 to 70	29 to 73
Sex		
Male	9	12
Female	23	19
Prior chemotherapy		
none	4	9
1	6	13
2	7	8
3	5	
4	2	
> 5	7	1
Tumor Type		
Pancreas	7	4
Breast	10	5
Colon	7	6
Esophageal	4	1
Other	1	6
Lung	3	9
Bone Metastasis		
No	21	23
Yes	5	6
Yes, disease in flat bones	6	2
Metastatic Disease		
No	11	4
One site	12	13
Multiple sites	9	14
Prior use of Mitomycin	8	5

**Table 2:** Distribution of Grade of Thrombocytopenia by Source and Dose of RNA Fragments

E. coli RNA fragments				
Grade/Dose	20 mg	40 mg	60 mg	80 mg
1	1		1	2
2	3	7	12	6
3	6	9	4	8
4	0	0	0	0
**Yeast RNA fragment**				
**Grade/Dose**	**20 mg**	**40 mg**	**60 mg**	**80 mg**
1	1	3	2	3
2	4	2	6	3
3	3	5	4	8
4	1	1	0	0

#### Yeast RNA Fragment Arm

Following the completion of the E. coli trial, forty patients signed consent forms to participate in the yeast RNA fragment arm. Nine consented patients did not participate in the trial; patients dropped out due to disease progression that required a change in treatment regimen after signing the consent form or they had become too fragile for treatment. Thirteen patients had no history of anti cancer therapy and only two patients had undergone five or more chemotherapy regimens. The treated patients were exposed to CIT agents such as, taxotere (N = 9), carboplatin (N = 9), cisplatin (N = 6), taxol (N = 6); and four patients had prior exposure to mitomycin. The median time between diagnosis of disease and participation in the trial was 39 weeks (0 to 296 weeks). All but two patients had metastatic disease. Eight patients had metastatic disease to the bone and two of these patients had metastatic disease to the flat bones that could affect the production of platelets (Table 1). The prevalence of Grade 3 or Grade 4 thrombocytopenia at the nadir prior to baseline or dose escalation of the RNA fragments was 43% (20/46 treatment cycles) and 4.3% (2/46) respectively (Table 2).

### Dose Response Relationships between Chemoprotectant Dose and Platelet Responses

#### E. coli RNA Fragment Arm

These patients underwent treatment protocols that contained one or more myelosuppressive agents. The most common myelosuppressive agents were carboplatin (n = 11), cisplatin (N = 6), Docetaxel (N = 6), Mitomycin (N = 5) and premetrexed (N = 5). All but nine patients used regiments that had multiple CIT inducing agents.

**Figure 1:**
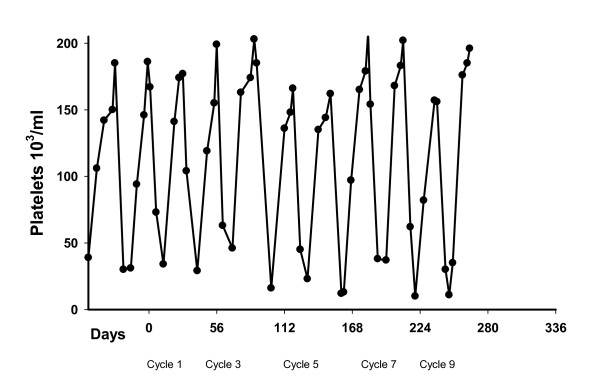
**Breast Cancer Patient with Metastatic Disease to Flat Bones with Two Prior Chemotherapy Regimens--Current Regimen is Doxorubicin**.

This clinically heterogeneous group of patients likely had a wide distribution in bone marrow reserve. Consequently, we examined whether there were any differences in the distribution of platelet counts at the time of first administration of RNA fragments or at dose escalation of the RNA fragments at day 1 of chemotherapy. There was no statistically significant difference in the platelet count by E. coli RNA fragment dose. The platelet levels one day 1 of chemotherapy were 162000/ml (treatment cycle number = 12), 177,500/ml (treatment cycle N = 25), 160000/ml (treatment cycle N = 21), and 187,000/ml (treatment cycle N = 47) for 20 mg, 40 mg, 60 mg and 80 mg respectively (Kruskal-Wallis P = 0.086). All of the patients on E. coli RNA fragments showed an oscillation in platelet counts where the platelet levels declined after the administration of chemotherapy and recovered during the rest phase of the treatment cycle, Figure 1. There was no statistically significant difference in the median time to chemotherapy induced platelet nadirs by RNA fragment dose (Kruskal-Wallis P = 0. 841). There was no statistically significant difference in the platelet counts in chemotherapy induced nadirs by fragment dose, or in the prevalence of nadir levels less than 25000/ml, which was 16.6%, 21%, 10%, and 24% for 20, 40, 60, and 80 mg levels respectively. The prevalence rate for nadir levels greater than 80000/ml, was 8.3%, 33%, 20%, and 28%, respectively for 20, 40, 60, and 80 mg indicating the relative failure of the 20 mg dose. This trend was a statistically significant (p < .05). Finally, there was a statistically significant shortening in time from chemotherapy induced nadir to attaining recovery platelet level of 80000/ml or more from a median recovery time of eight days for the 20 mg dose to a median time to recovery of 6 or 7 days for the other doses (Kruskal-Wallis P = 0.044).

Finally, there were several patients who exhibited slowed rates of platelet recovery following removal of the E. coli RNA fragments.

#### Yeast RNA fragment Arm

All but seven patients underwent regimens that used multiple CIT inducting agents. The most common CIT agents used in the regimens were Carboplatin (N = 7), cisplatin (N = 7), and mitomycin (N = 7). Patients participating in the yeast RNA trial were a clinically heterogeneous group. We evaluated whether there were any differences in the distribution of platelet counts at the time of first administration of RNA fragments or at dose escalation of the yeast RNA fragments at day 1 of chemotherapy. The median platelet levels on chemotherapy day 1 were 124,500/ml (treatment cycle N = 11), 157000/ml (treatment cycle N = 18), 143000/ml (treatment cycle N = 13), and 128500/ml (treatment cycle N = 31) for 20, 40, 60, and 80 mg respectively (Kruskal-Wallis p = 0.334).

There was no statistically significant difference in the time to chemotherapy induced platelet nadir by RNA fragment dose level (Kruskal-Wallis P = 0.824) nor a statistically significant relationship in the platelet nadir counts by RNA dose; the median nadir levels were 42000/ml, 66000/ml, 45000/ml, and 53000/ml for 20, 40, 60, and 80 mg dose levels respectively. Unlike the E. coli data, there was no statistically significant shortening in the time from chemotherapy induced nadir to a recovery platelet level of 80000; median platelet recovery times were 12, 7, 12, and 11.5 days for 20, 40, 60, and 80 mg doses (Kruskal-Wallis P = 0.573).

**Figure 2:**
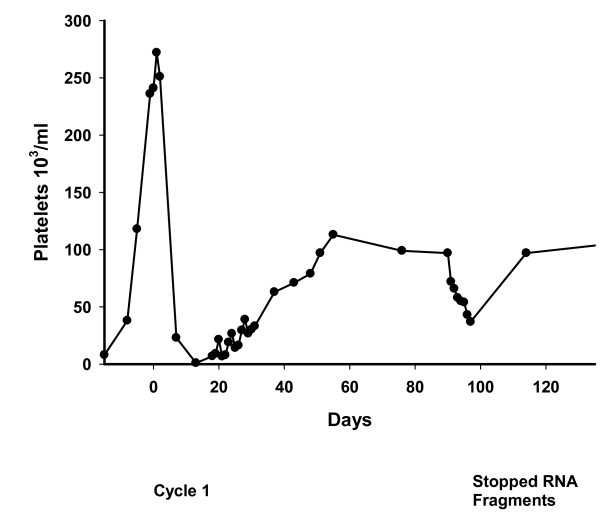
**Metastatic Lung Cancer Patient (Long Bones/Lymph Nodes) Failed Three Prior Regimens and Undergoing Ifosamide/Doxorubicin/Decabazine**.

Unlike the patients on E. coli fragments, seven patients using yeast RNA fragments failed to demonstrate an oscillation in platelet counts, Figure 2. In these cases platelet levels declined after the administration of chemotherapy but either did not recover or recovered very slowly during the rest phase of the treatment cycle.

### Dose Response Relationships

The dose response relationships with E. coli RNA fragments chemotherapy induced platelet nadir levels were evaluated at baseline or the initial dose escalation of RNA fragments. The median nadir platelet levels ranged from 46000 at 20 mg to 55000 for the 80 mg, which was not statistically significant (Kruskal-Walis two sided p = 0.581). As administered in this study, increasing the dose of E. coli RNA fragments do not affect chemotherapy's capacity to depress platelet levels. Similarly, there was no statistically significant dose response relationship between nadir levels and increasing doses of yeast RNA fragments (Kruskal-Walis P = 0.262).

**Figure 3:**
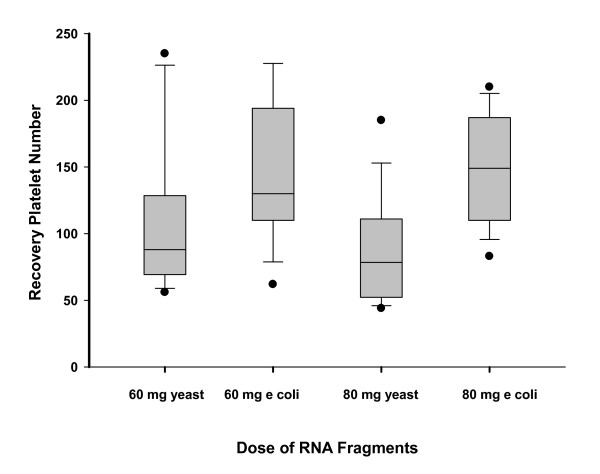
**Comparison in Platelet Recovery Levels following CIP Nadirs by Type of RNA Fragments**.

There was a significant relationship between recovery platelet levels and increasing doses of E. coli RNA fragments at the baseline or initial dose escalation of RNA fragments. Platelet recovery levels were measured between five to eight days following chemotherapy induced nadirs. The median recovery platelet counts rose from 110000/ml at 20 mg to 130000/ml at 60 mg and 149000/ml at 80 mg (Kruskal-Wallis two sided p = 0.0149). There was no observed relationship between dose of yeast RNA fragments and the median number of platelets five to eight days post chemotherapy induced nadir (Kruskal-Wallis P = 0.31). There was a statistically significant difference in Platelet recovery levels by type of RNA fragments. E. coli Fragments at the 60 and 80 mg levels exhibited a statistically significant higher recovery level five to eight days after chemotherapy induced nadir than Yeast RNA fragments (Kruskal-Wallis P < 0.001, Figure 3).

There were twelve patients who were treated with 60 mg of E coli RNA fragments that were dose escalated to the 80 mg dose. We, therefore, compared the efficacy of the 80 mg dose to the 60 mg dose on the patients' nadir levels. The platelet counts at the 60 mg dose ranged from 17000 to 72000 (median 45500) in these twelve patients, while their nadir platelet counts following dose escalation to 80 mg dose of E coli RNA fragments ranged by 16000 to 129000 (median 65,500). A pair-wise comparison found that nine patients had higher nadirs at the dose escalated 80 mg dose (platelet counts increased by 17,000 to 109,000) compared to their nadirs at 60 mg, three patients had lower platelet counts following dose escalation to 80 mg dose (platelet counts decreased from 7,000 to 30000). Overall, there was a statistically significant difference between these two groups (Wilcoxon Signed Test P = 0.011).

### Stratification by Chemotherapy

All of the patients recruited to this trial had the potential to develop clinically concerning chemotherapy induced low platelet levels. Among the wide variety of chemotherapeutic agents used in this trial, Platinum based agents and Mitomycin were the most efficient inducers of CIT. In both E. coli RNA and Yeast RNA, there was no statistically significant difference in the platelet number five to eight days after chemotherapy induced nadir between treatment protocols that used platinum based agents or mitomycin and those that did not, see table 3 and 4.

**Table 3:** The Relationship between Therapeutic Agents and Platelet Recovery Following Nadir in E. coli

RNA Fragments	Dose	Chemotherapy	Patient Number	Median Platelet Counts	Range	Kruskall Wallis
E. coli	60	Non mito, non Platinum	11	163	90 to 257	0.09
		Carboplatin	2	122.5	118 to 127	
		Cisplatin	2	95	62 to 129	
		Mitomycin C	1	62	62	
E. coli	80	Non mito, non Platinum	10	137.5	83 to 210	0.3
		Carboplatin	2	156	110 to 202	
		Cisplatin	1	173	173	
		Mitomycin C	2	168	149 to 187	

**Table 4:** The Relationship between Therapeutic Agents and Platelet Recovery Following Nadir in Yeast

RNA Fragments	Dose	Chemotherapy	Patient Number	Median Platelet Counts	Range	Kruskall Wallis
Yeast	60	Non mito, non Platinum	5		21-235	
		Carboplatin	4		56-130	
		Cisplatin	2		101-206	
		Mitomycin C	1		101	
Yeast	80	Non mito, non Platinum	6		57-244	
		Carboplatin	7		33-121	
		Cisplatin	1		185	
		Mitomycin C	3		40-90	

### Clinical Sequelae and Platelet Support

The primary clinical goal of anti-chemotherapy induced thrombocytopenia is the avoidance of delays in treatment and reductions in the dose of chemotherapy. Four patients experienced a treatment delay or reduction in chemotherapy dose in the yeast fragment arm of the trial, but no patient using E. coli, RNA fragments had an unplanned dose reduction or delay.

The other important clinical goal for anti thrombocytopenia is the reduction and prevention of platelet transfusions. Four patients had platelet transfusions while taking E. coli RNA. At the 20 mg dose two patients had platelet transfusions. One of these patients had a concurrent GI bleed related to tumor. At the 40 mg level, two patients had platelet transfusions and one experienced a cerebral bleed. There were no platelet transfusions in patients dosed at the 60 or 80 mg E coli, RNA fragments.

Three patients required platelets when they came off trial or failed to use E. coli fragments. Two patients had platelet transfusions 30 days and 16 days after coming off E. coli RNA fragment trial while the third experienced bleeding gums and required one unit of platelets after inadvertently failing to take RNA fragments for one treatment cycle. Interestingly, this patient underwent multiple cycles of Myelosuppressive Therapy with E. coli RNA fragments before and after this unprotected cycle without incident. These data are consistent with the hypothesis that E coli RNA fragments accelerate the recovery in platelet counts following chemotherapy-induced thrombocytopenia.

Four of the thirty-one patients on the yeast RNA arm of the trial had platelet transfusions; one transfusion at 40 mg, three at 60 mg, and one at 80 mg doses. No thrombocytopenic related symptoms were reported among these patients.

## Discussion and Conclusions

The emergence of Grade 3 or 4 thrombocytopenia in a cancer patient correlates with a significant worsening in the patient's prognosis [3]. Among the other agents in development for treatment of CIT the mechanism of action of short chain prokaryotic RNA fragments is unique. Both cell division and differentiation are required for platelet proliferation, and DNA replication is the primary step. DNA synthesis depends on special naturally produced short chain RNA fragments as primers for DNA polymerase [13-15]. These natural primers might very well derive from the RNA of intestinal E. coli absorbed fragmented by native RNAases. In chemotherapy patients, in whom damage to cells of the intestinal lining is a common side effect, the E. coli RNA fragments developed by Beljanski appear to fulfill this primer function when administered exogenously. In vitro studies show that these RNA fragments act as primers for DNA synthesis in the progenitor cells that give rise to platelets [5,6].

By study criteria, all trial participants had developed grade 1, 2, or 3 CIT before being administered the study drug. Moreover, all of these patients had experienced extensive prior history of exposure to myelosuppressive drugs and approximately a third of these patients had metastatic disease to the bone marrow where platelets are produced thereby identifying them to be at high risk for Grade 3 or 4 CIT.

At all doses, patients receiving E. coli RNA fragments showed a recovery in platelet numbers to 80000/ml by eight days following chemotherapy induced nadir. Moreover, the platelet recovery levels following chemotherapy induced nadir rose in a dose dependant manner from 110000 at the 20 mg dose to 149000 at the 80 mg dose. No patient had an unplanned dose reduction or treatment delay nor did any E. coli RNA fragment patient treated at the 60 and 80 mg dose level need a platelet transfusion although four patients had platelet transfusions at the lower doses. After discontinuing E. coli RNA fragment therapy, there appeared to be a decline in the recovery rate of platelet numbers in five patients, which suggest the RNA fragments were involved in accelerating platelet recovery in these patients.

Seven patients using yeast RNA fragments failed to show a rebound in platelets numbers following chemotherapy induced nadir. Four yeast RNA fragment patients experienced dose reductions or delays in their treatment. There was no evidence of a dose response relationship between yeast RNA and platelet recovery levels following chemotherapy induced nadirs and there were platelet transfusions at the higher three yeast RNA dose levels (40, 60, and 80 mg). There was no relationship found between the specific chemotherapy agents and platelet recovery levels. These data indicate that RNA fragments manufactured from yeast are less effective than RNA fragments manufactured from E. coli in accelerating the rate of platelet recovery following CIT.

Limitations of this trial include inconsistent timing and frequency of platelet counts. Prospectively collected data on patients provided outcomes regarding administration of agent and side effects but did not include the signs and symptoms of thrombocytopenia such as bleeding or petechiae. There was a lack of consistency of documentation in the patient diary and not all diaries were presented to the data management team. All efforts were made to capture missing data from the medical record and personal contact with patient. This was also non-ideal patient population to test the effectiveness of an anti-thrombocytopenia drug. A significant fraction of these patients had already failed one or more regimens, some had used agents that induce long term bone marrow damage while others had metastatic disease to the bone. Many patients in this trial experienced tumor progression which resulted in their being terminating from the trial. In this challenging patient population, we found data that a 80 mg dose of E coli RNA fragments appear to accelerate platelet recovery in patients undergoing chemotherapy and we have data suggesting the 80 mg E coli RNA may ameliorate chemotherapy induced thrombocytopenia. This preliminary data supports the development of further clinical trials designed to measure the efficacy of E. coli RNA fragments in the management of CIT among patients with advanced cancer.

## Competing interests

John L Hall PhD is employed by Natural Source International^® ^as Director of Research.

## Authors' contributions

RDL, MD, and JFG participated in concept, design, data collection, data analysis, data interpretation and writing. JLH participated in concept, design, data interpretation and provided the details of the mechanism of action of the RNA fragments. CGL participated in concept, design and general oversight of the study. All authors read and approved the final manuscript.

## Pre-publication history

The pre-publication history for this paper can be accessed here:

http://www.biomedcentral.com/1471-2407/10/565/prepub
